# The Triple Procedure in Patients with Congenital Aniridia

**DOI:** 10.3390/jcm13216619

**Published:** 2024-11-04

**Authors:** Bogumił Henryk Wowra, Olga Łach-Wojnarowicz, Marzena Wysocka-Kosmulska, Dariusz Dobrowolski, Edward Wylęgała

**Affiliations:** 1 Chair and Clinical Department of Ophthalmology, Faculty of Medical Sciences in Zabrze, Medical University of Silesia in Katowice, 65 Panewnicka Street, 40-760 Katowice, Poland; olga.lachwojnarowicz@gmail.com (O.Ł.-W.); m.wysocka554@gmail.com (M.W.-K.); dardobmd@wp.pl (D.D.); wylegala@gmail.com (E.W.); 2 Department of Ophthalmology, District Railway Hospital, 65 Panewnicka Street, 40-760 Katowice, Poland

**Keywords:** congenital aniridia, cornea transplantation, cataract extraction

## Abstract

**Background:** Aniridia is a rare panocular, bilateral, and congenital disease characterized by complete or partial iris hypoplasia and foveal hypoplasia, leading to decreased visual acuity and nystagmus. AAK, also referred to as aniridic keratopathy, manifests as corneal surface damage, epithelial thinning or loss, inflammation with immune cell infiltration, vascularization, and chronic progressive opacification. **Methods:** Twenty-one eyes in eighteen patients with aniridia underwent the triple procedure for visual rehabilitation. Subjects with stromal scarring with mild limbal deficiency were qualified for surgery. The majority of them developed stage II (15), and a few of them had third-degree (6) aniridic keratopathy. **Results:** The mean patient age was 38.4 ± 8.8. Visual acuity after one year of observation ranged from 0.4 in two eyes to 0.2 in nine eyes to below 0.1 in ten eyes. In the second year, VA remained at the same level in 13 patients (72.2%). In the third year, four patients (22.2%) experienced recurrence of AAK. **Conclusions:** A majority of the ARK cases (72.2%) had a graft providing useful vision for the patient 2 years after corneal transplantation, but the visual gain was modest at best. Longer follow-up time is required to evaluate functional graft outcomes.

## 1. Introduction

Aniridia is a rare and complex ocular disorder characterized by incomplete or total iris underdevelopment and foveal hypoplasia, resulting in reduced visual acuity and often accompanied by nystagmus. Its prevalence is estimated to range from 1 in 40,000 to 1 in 100,000 individuals, with no significant association with race or gender [1,2].

The condition is primarily inherited in an autosomal dominant manner, with high penetrance but variable expressivity even within affected families. The majority of cases are caused by mutations in the paired-box gene 6 (PAX6), although sporadic cases also occur. Notably, aniridia can present either as an isolated ocular malformation or as part of a syndrome, such as WAGR syndrome (Wilms tumor, aniridia, genitourinary anomalies, and developmental delay) or Gillespie Syndrome (partial aniridia, non-progressive cerebellar ataxia, and intellectual disability) [3].

The mechanisms underlying ocular alterations in aniridia are not fully understood. The role of PAX6 in both the optic vesicle and lens development suggests a complex interplay between these structures. While previous research suggested a predominant role of lens dysfunction in aniridia, recent studies have highlighted the importance of PAX6 expression in the optic vesicle for maintaining contact with the lens epithelium and regulating cell survival. Studies in mouse chimeras with PAX6 mutations suggest that alterations in the lens may drive secondary changes in other ocular tissues, such as the iris and cornea. This observation raises the possibility of targeted gene therapy to correct lens abnormalities, potentially improving overall ocular function and vision in individuals with aniridia. By supplementing existing diagnostic methods with prenatal genetic testing, clinicians can identify affected embryos and potentially intervene with gene therapy to address lens abnormalities early in development. This approach holds promise for improving clinical outcomes and preventing ocular degeneration and blindness associated with aniridia [4,5,6].

One of the most common complications associated with aniridia is aniridia-associated keratopathy (AAK), which manifests as corneal opacification. Additionally, individuals with aniridia are at increased risk of developing glaucoma and cataracts. Despite significant advances in understanding the genetic basis of aniridia, the precise molecular mechanisms underlying its various pathological features remain unclear. Efforts to improve treatments for AAK focus on innovative approaches, including not only gene therapy using adeno-associated virus (AAV) delivery but also in vivo gene editing with CRISPR/Cas9 and nonsense mutation suppression. Drug repurposing also shows promise. Gene therapy aims to correct corneal genetic defects, requiring precise PAX6 regulation to avoid harmful overexpression. Elizabeth Simpson’s team [7] has created vectors with “MiniPromoters” for targeted delivery to corneal cells in aniridia mouse models, highlighting potential therapeutic applications. CRISPR/Cas9 offers precise gene editing options, creating mouse models that mimic human aniridia and paving the way for future human treatments, with a focus on ensuring safety [8]. Pharmacological strategies include nonsense suppression drugs like Ataluren, which facilitates full protein synthesis by bypassing premature stop codons. Though promising, this approach faced challenges in trials due to patient variability. Local delivery may enhance efficacy [9]. Modulating Pax6 expression through MEK inhibitors shows promise in enhancing Pax6 levels, with drugs like Duloxetine and Ritanserin being identified as potential agents to boost Pax6, opening new treatment avenues [10]. These emerging therapies, especially if combined with repurposed drugs and Pax6-enhancing agents, could significantly improve aniridia management, benefiting the over 7000 patients with congenital aniridia in the EU by offering rapid and accessible treatment options. Further research into the molecular and physiological origins of aniridia-related complications is essential for developing targeted therapies and improving clinical management strategies for affected individuals. By unraveling the complexities of this condition, we can enhance our ability to diagnose, treat, and potentially prevent the debilitating consequences of aniridia [11,12].

### 1.1. Aniridia Symptoms

Aniridia presents a constellation of symptoms predominantly affecting the eyes. Foveal and iris hypoplasia, along with aniridia-associated keratopathy (AAK), represent the most prevalent phenotypic anomalies of congenital aniridia, observed in 80–100% of cases. Individuals with aniridia commonly exhibit reduced visual acuity and color vision, often experiencing blurred or unclear vision. Nystagmus is frequently observed, further compromising visual stability. Poor tear film quality and low tear production result in dry eye disease (DED), which is experienced by approximately half of the patients [13]. Cataracts and glaucoma are other hallmarks of aniridia which contribute to visual impairment. In addition to affecting the eyes, congenital aniridia also impacts various other organs, including the brain, gut, pancreas, and olfactory and auditory systems [14,15]. Additionally, aniridia has been associated with other conditions such as obesity and narcolepsy [16].

### 1.2. Aniridic Keratopathy

AAK, also referred to as aniridic keratopathy, manifests as corneal surface damage, epithelial thinning or loss, inflammation with immune cell infiltration, vascularization, and chronic progressive opacification [11]. The progression of keratopathy in aniridia begins with a healthy limbal border, appearing complete and intact, similar to a normal corneal limbus under slit lamp examination. As the disease advances, there is an initial breakdown of the limbal border, accompanied by the early encroachment of conjunctival tissue across the limbus. However, initially, the conjunctival tissue remains in close proximity to the limbal area. Subsequently, AAK involves further ingrowth of conjunctival tissue into the peripheral and paracentral cornea. This stage is often marked by the presence of a vascular pannus or clouding due to vessel ingrowth, sometimes termed conjunctivalization. Progressing sequentially, the central part of the cornea becomes affected by vascularization and the involvement of the corneal stroma. The most severe grade represents an advanced, end-stage AAK, where the cornea undergoes total conjunctivalization and vascularization, resulting in its transformation into an opaque, thick, and irregular structure [17]. The clinical classification of congenital aniridic keratopathy is presented in Table 1.

The pathogenesis of AAK is multifactorial and not yet fully understood. However, several mechanisms have been proposed to contribute to its development. AAK symptoms resemble those observed in conditions where limbal stem cells (LSCs) are absent from the corneal periphery due to disease or injury. The dysfunction or deficiency of these stem cells can lead to impaired corneal epithelial turnover and repair, contributing to the development of AAK [18,19]. There are also hypotheses proposing that the PAX6 gene regulates many of the physiological and biological factors associated with AAK [20]. Aniridia can affect the composition and integrity of the corneal basement membrane, which plays a crucial role in maintaining corneal transparency and homeostasis. Disruption of the basement membrane can lead to corneal epithelial abnormalities and increased susceptibility to damage. Additionally, persistent inflammation, possibly triggered by the underlying genetic mutation or secondary to corneal surface irregularities, can contribute to the progression of AAK. Inflammatory mediators released during the inflammatory process can further disrupt corneal structure and function [21]. Thus, further comprehensive research is warranted to conclusively validate these theories.

**Table 1:** Clinical classification of congenital aniridic keratopathy by Lopez-Garcia et al. [22].

Stage	Clinical Signs and Symptoms
**Grade 0**	Absence of signs and symptoms, but cytologic changes.
**Grade 1**	Pannus less than 1 mm. Fine epithelial staining with fluorescein-epiphora. Occasional epithelial defects.
**Grade 2**	Circumferential pannus with free visual axis. Photophobia, epiphora, lacrimal instability, and epithelial defects (two or more episodes in the last six months).
**Grade 3**	Stromal opacity with deposits and fibrosis affecting the corneal center. Chronic inflammation, marked neovascularization, and chronic epithelial defects.

### 1.3. Combined Procedures of Keratoplasty and Cataract Surgery

**Figure 1:**
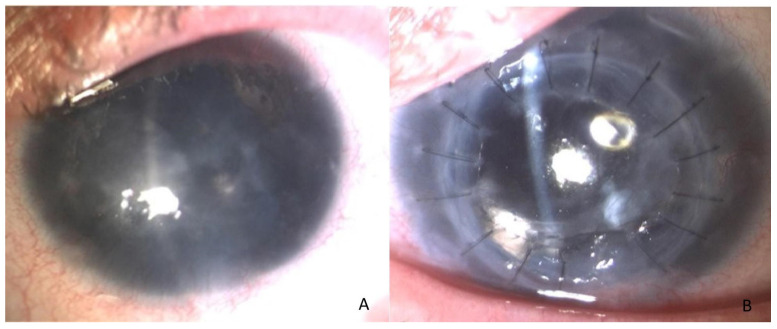
Patient before (**A**) and after (**B**) triple procedure.

The triple procedure involves penetrating keratoplasty, extracapsular cataract extraction, and posterior chamber lens implantation (Figure 1). This surgical combination is used to treat patients with significant corneal pathology and cataracts, aiming to restore vision in one operation [23]. Keratoplasty, or corneal transplantation, is one surgical approach used to improve vision in patients with AAK. However, the procedure is complex due to the immunocompromised status of the ocular surface and the high risk of graft rejection and failure. Although lamellar surgery is often favored over penetrating keratoplasty to reduce rejection risks, the success rates remain low due to complications like neovascularization and wound healing problems. Alternatively, keratoprosthesis, such as Boston type I, serves as a last resort for severe cases, offering non-opacifying optical solutions but with significant side effects and complications.

The integration of cataract surgery with intraocular lens (IOL) implantation and penetrating keratoplasty (PKP) for patients with concurrent corneal disease and cataracts was first outlined in 1976 [24]. Given the cornea’s accessibility and replaceability, it stands as a promising target for therapies aimed at improving vision in aniridia. In instances where corneal diseases, frequently accompanied by cataracts, are amenable to keratoplasty, a triple procedure encompassing penetrating keratoplasty, extracapsular lens extraction, and IOL implantation could be contemplated. While this approach offers benefits, such as streamlined treatment, rapid visual rehabilitation, and minimal additional endothelial injury, it also carries risks. Open-sky surgery may entail uncontrolled vitreous pressure, posterior capsule rupture, challenges with IOL implantation, and, in the worst-case scenario, expulsive hemorrhage. While a sequential procedure for performing cataract surgery after keratoplasty allows for the accurate estimation of intraocular lens (IOL) power, it comes with several disadvantages. These include slow visual rehabilitation and the risk of endothelial damage during cataract surgery [25]. Therefore, in cases of corneal opacity accompanied by significant cataracts, the preferred treatment option is often the triple procedure.

Studying the outcomes of corneal transplantation in aniridia-related keratopathy (ARK) is of paramount importance due to the complexities and challenges associated with this condition, particularly when surgical intervention is necessary. ARK is relatively uncommon, and there is a scarcity of studies available on the surgical outcomes in this patient population. Consequently, for clinicians tasked with managing patients with ARK experiencing poor and deteriorating visual acuity, navigating treatment decisions can be particularly daunting.

The rarity of ARK and the limited data on surgical outcomes underscore the need for further research to better understand the efficacy and limitations of corneal transplantation in this context. By elucidating the factors influencing surgical success, such as patient selection criteria, surgical techniques, and postoperative management protocols, clinicians can optimize treatment strategies and improve patient outcomes. Moreover, comprehensive and multidisciplinary care approaches are essential for managing the complexities of ARK effectively. This may involve collaboration between ophthalmologists, corneal specialists, geneticists, and other healthcare professionals to address both ocular and systemic manifestations of the disease. Ultimately, advancing our understanding of the surgical outcomes in ARK is crucial for guiding clinical decision-making and improving the quality of life of individuals affected by this challenging condition. By fostering ongoing research and collaboration within the medical community, we can strive to optimize treatment approaches and enhance visual outcomes for patients with ARK. The aim of our study was to evaluate the ophthalmological results in patients with aniridia after using the triple procedure for the treatment of AAK and cataracts in order to determine the efficacy and safety of this procedure in long-term follow-up.

## 2. Materials and Methods

The Polish cohort of patients with aniridia totals around 300 individuals, with approximately 35% attributed to de novo mutations, while aniridia associated with WAGR syndrome represents approximately 1.8%. AAK is usually diagnosed in the 2nd decade of life, while the first therapeutic procedures are performed in the 3rd decade, including cataract removal performed in the 3rd or 4th decade. Approximately half of the patients develop glaucoma.

### 2.1. Patients’ Characteristics

Twenty-one eyes in eighteen patients with aniridia underwent the triple procedure for visual rehabilitation. Surgeries were performed in two Silesian ophthalmology clinics. Subjects with stromal scarring with mild limbal deficiency were qualified for surgery. The majority of them developed stage II (15); a few of them had third-degree (6) aniridic keratopathy. Advanced keratopathies with total opacification were referred for limbal surgery or keratoprostheses. All cases included partial conjunctival ingrowth with areas of present corneal epithelium. The main inclusion criterium was central involvement due to stromal scarring, deep vasculature, or conjunctival ingrowth over the central cornea. Four eyes experienced central ulcers that healed with amniotic membrane grafts. Treatments were administered to patients with severely reduced visual acuity, measured by counting fingers (CFs), and very poor corneal transparency, rendering them unsuitable candidates for cataract surgery. Fourteen patients were pre-treated with superficial keratectomy; four eyes underwent a previous COMET procedure (cultivated oral mucosal epithelial transplantation); and three eyes were without any previous surgical intervention.

### 2.2. Triple Procedure Data

Axial length was assessed using ultrasound A-scan (Zeiss IOL Master 500, Zeiss, Oberkochen, Germany). Manual keratometry was attempted for all eyes; however, keratometry (K) values were incorporated into intraocular lens (IOL) power calculations only if the mires were clear. In cases where manual keratometry was unsatisfactory, corneal topography was utilized to ascertain the mean simulated K value. If the keratograph mires were excessively distorted, a standard K reading (44.0 diopters [D]) was employed for the IOL power calculation.

All triple procedures were performed by the same surgeon. In both techniques, systemic anesthesia was used.

The host cornea from 7.0 mm to 8.0 mm was trephined with a disposable manual trephine (Bausch & Lomb, Bridgewater, NJ, USA). A beer can capsulotomy was created with a capsulotome. Open-sky ECCE was performed by gentle nucleus delivery followed by manual I/A with a Simcoe I/A cannula. A 3-piece IOL was implanted in the capsular bag. The donor corneal button, which was 0.5 mm larger in diameter than the recipient bed, was sutured with 16 interrupted or 16-bite continuous 10-0 monofilament nylon.

Patients were observed with the standard postop regimen: 1–2 weeks, the first month, every 3 months in the first year, and every 6 months thereafter. Dexamethasone was administered every three hours for the 1st week, TID for 2 weeks, and BID thereafter. A local antibiotic (3rd generation fluorochinolone) was administered four times a day for ten days. The general antibiotic ciprofloxacin 500 mg BID was offered for 5 postop days.

Graft transparency, in terms of AAK neovascularization recurrence in four quadrants and visual acuity (VA), was analyzed in long-term follow-up.

## 3. Results

The mean age of the patients in our study was 38.4 years, with a standard deviation of 8.8 years. After one year of observation, visual acuity (VA) varied among patients, with two patients achieving a VA of 0.4, nine eyes of 0.2, and ten eyes of below 0.1. In the second year, VA remained stable in 13 patients (72.2%). However, eight patients experienced conjunctival ingrowth, leading to a decrease in VA in the range of 0.01 to 0.04.

By the third year, four patients (22.2%) exhibited recurrence of aniridia-associated keratopathy (AAK), with one patient classified as grade 1 and three patients as grade 2. Two patients were subsequently referred for keratoprosthesis due to the severity of their condition. Additionally, conjunctival ingrowth extended to involve two quadrants in two eyes, three quadrants in four patients, and two patients developed total limbal stem cell deficiency (LSCD).

## 4. Discussion

Aniridia is a rare congenital ocular disorder, primarily resulting from PAX6 gene mutations [1], that significantly impacts visual function. Characterized by incomplete or absent iris development and foveal hypoplasia, and associated with systemic abnormalities, its clinical complexity poses numerous therapeutic challenges [26]. The triple procedure—penetrating keratoplasty, extracapsular cataract extraction, and intraocular lens implantation—has been a promising surgical approach for managing patients with aniridia-associated keratopathy (AAK) and cataracts [27]. This combined approach aims to restore corneal clarity, correct refractive errors, and improve visual acuity.

In our study, we evaluated the outcomes of this triple procedure on a cohort of aniridia patients, highlighting both its potential benefits and limitations. The penetrating keratoplasty (PKP) component addresses the corneal opacification that typically accompanies AAK, where vascularization and conjunctivalization compromise visual transparency. However, PKP carries significant risks in these patients due to the compromised limbal stem cell function and the high propensity for graft rejection and recurrence of neovascularization [28]. Moreover, the complexity of this procedure is heightened in cases of significant conjunctival ingrowth, which may exacerbate graft failure [29].

At the two-year follow-up, 72.2% of the patients’ grafts available for evaluation were functioning, indicating a positive trend toward improved visual acuity compared to pre-surgery levels. Five patients were lost at the two-year follow-up. However, it is noteworthy that the recurrence of AAK was not uncommon (22.2%), underscoring the need to carefully consider other potential causes of severe vision loss beyond corneal opacities in ARK cases before proceeding with corneal transplantation. This emphasizes the complexity of managing aniridia and the importance of comprehensive preoperative assessment and ongoing postoperative care to optimize visual outcomes.

Cataract extraction and posterior chamber intraocular lens (IOL) implantation are critical in addressing cataracts, another common manifestation in aniridia patients [30]. While the procedure can significantly enhance visual rehabilitation, its integration with keratoplasty (open-sky cataract surgery) introduces additional risks, such as posterior capsule rupture and uncontrolled vitreous pressure [31]. Despite these challenges, the simultaneous approach often streamlines the rehabilitation process, offering a quicker recovery of vision. However, postoperative complications, including the recurrence of AAK and limbal stem cell deficiency, remain significant hurdles that can compromise long-term visual outcomes [32].

Postoperatively, graft transparency was achieved in many cases, though complications such as conjunctival ingrowth and recurrent AAK were observed [2], emphasizing the need for vigilant follow-up and potential adjunctive therapies to sustain long-term graft survival and visual function. Recent innovations, including gene therapy and regenerative approaches, hold promise for addressing underlying genetic deficits, particularly through targeted PAX6 gene therapy [33] and the potential use of CRISPR/Cas9 [34,35]. These molecular strategies aim to address both the epithelial abnormalities in the cornea and the lens dysfunction at the root of the disorder, offering a more targeted and potentially curative treatment pathway [36].

Additionally, pharmacological advancements, including nonsense mutation suppression drugs [37] and MEK inhibitors aimed at modulating PAX6 expression [38], represent promising future treatment modalities that could complement surgical interventions. These emerging therapies, combined with the procedural advances seen in the triple procedure, underscore a shift toward a more holistic approach to aniridia management, where genetic correction, pharmacotherapy, and surgical techniques are integrated [39,40].

Artificial iris implantation, particularly using sutured posterior chamber black diaphragm intraocular lenses (IOLs), has been explored as a potential treatment for patients with aniridia or severe iris defects. However, recent studies have highlighted significant complications associated with this technique. These complications include the development or worsening of glaucoma and the exacerbation of aniridia-associated keratopathy (AAK), a condition where the corneal surface becomes compromised. As a result, while this option offers cosmetic and functional benefits, the risks make it a less ideal choice, especially in complex cases [41].

This study is constrained by a limited number of cases, which undermines its statistical power to effectively compare various surgical methods, identify significant risk factors, and assess their respective impacts on graft survival and visual outcomes. This limitation emphasizes the need for larger sample sizes or multicenter collaborations to provide more comprehensive insights into anterior lamellar keratoplasty (ALK) procedures.

Despite this limitation, this study yields valuable insights into the clinical practices of Polish corneal surgeons. It demonstrates their adherence to evidence-based recommendations by exercising caution when selecting ALK as a treatment modality. By reserving corneal transplantation as a last resort, these surgeons prioritize conservative approaches that aim to preserve corneal integrity and function whenever possible. This approach not only reflects a commitment to patient-centered care but also underscores the importance of judicious decision-making in the management of corneal pathologies.

While the triple procedure presents a viable surgical option for managing AAK and cataracts in aniridia patients, it is not without challenges. The high incidence of postoperative complications, particularly conjunctival ingrowth and recurrent keratopathy [42], necessitates careful patient selection, comprehensive preoperative planning, and diligent postoperative management. Future advancements in gene therapy and pharmacology may offer significant improvements, potentially alleviating the need for invasive surgeries by targeting the genetic and molecular underpinnings of the disease [43].

## 5. Conclusions

This study faced several limitations, particularly due to its small sample size, which restricts its statistical power. This limitation makes it challenging to generalize the findings to the broader population or effectively compare and evaluate the different surgical methodologies employed. Additionally, aniridia itself is a complex disorder that affects both ocular and systemic functions, resulting in a wide range of symptoms and outcomes among patients. This variability poses challenges when interpreting study results, as the methods used may not fully account for these diverse manifestations and their impact on patient outcomes. Moreover, while corneal transplantation showed significant short-term benefits in improving visual acuity, there was a notable recurrence rate of aniridia-associated keratopathy (ARK) among patients. This recurrence underscores the ongoing challenges in maintaining long-term visual improvement and suggests that additional strategies may be required to address this issue.

For future research, involving larger patient cohorts and conducting studies across multiple centers would provide more comprehensive insights into the efficacy of corneal transplantation and various surgical approaches for treating ARK, thus allowing for more robust and generalizable conclusions. It is also crucial to continue evaluating and refining surgical techniques and postoperative care protocols. By enhancing these methods, researchers and clinicians can aim to improve long-term outcomes for patients with ARK, reduce complications, and enhance quality of life. Furthermore, developing collaborative strategies involving ophthalmologists, geneticists, and other healthcare professionals is vital. These multidisciplinary approaches can effectively address both the ocular and systemic manifestations of aniridia, offering holistic care to those affected. Finally, further research into the genetic and molecular basis of aniridia is essential. By unraveling the complexities of the condition at a molecular level, researchers can develop targeted therapies that address the underlying causes of the disease, leading to more effective treatments and potentially better patient outcomes.

In conclusion, our study revealed promising outcomes for patients with aniridia-related keratopathy (ARK) who underwent corneal transplantation. More than half of the patients experienced significant improvements in visual acuity one year post-surgery and maintained a functioning corneal graft for up to two years after transplantation. Despite encountering complications, such as recurrent ARK in the graft, conjunctivalization, or stromal haze, the observed enhancement in visual acuity was substantial.

## Data Availability

The data used to support the findings of this study are included in the article. The data will not be shared due to third-party rights and commercial confidentiality.
